# Novel treatment paradigms for metastatic uveal melanoma

**DOI:** 10.1038/s41417-022-00558-y

**Published:** 2022-12-01

**Authors:** Sapna P. Patel, Steven C. Katz

**Affiliations:** 1 Department of Melanoma Medical Oncology, MD Anderson Cancer Center, Houston, TX USA; 2 Brown University Warren Alpert School of Medicine, Department of Surgical Oncology, 2 Dudley Street, Suite 470, Providence, RI USA

**Keywords:** Metastasis, Prostate cancer

Uveal melanoma (UM) is the most common primary intraocular malignancy, accounting for 85 to 95% of primary ocular malignancies and 3 to 5% of all melanoma cases [1]. UM arises from melanocytes within the uveal tract, which consists of the iris, ciliary body, and choroid. Definitive treatment of the primary tumor with radiotherapy or enucleation results in low rates of local recurrence. However, despite effective local control, metastatic disease occurs in more than 50% of patients [2]. Metastatic UM involves the liver in greater than 90% of cases and arises from hematologic spread [3]. Intrahepatic immunosuppression is a critical driver of malignant progression in the liver, and myeloid-derived suppressor cells are central to this pathologic state in patients with liver metastasis [4]. Myeloid-derived suppressor cells have specifically been implicated in the pathogenesis of UM liver metastases (LM) [4–6].

Metastatic disease has poor outcomes, with 1-year overall survival (OS) rates of 43% from the time of the original diagnosis [7]. For metastatic UM, treatment can be grouped into several categories, including liver-directed therapies, cytotoxic chemotherapy, immunotherapy, molecularly-targeted therapies, and epigenetic modifiers. Liver-directed therapies include resection, radiofrequency ablation, stereotactic radiotherapy, intralesional therapy, regional therapy and embolization. The use of regional chemotherapy with fotemustine and melphalan via intrahepatic artery infusion was compared with systemic chemotherapy without improvement in OS [8]. As immune-suppressive cells and pathways are critical drivers of disease progression in patients with liver metastasis from multiple malignancies [9], cytotoxic therapies that fail to address the immunologic defect in the liver may have limited success. Application of immuno-oncology agents through systemic infusion is theoretically appealing, but challenges remain with respect to the highly suppressive immune environment in the liver and effective delivery of immunotherapeutics to liver metastasis in the absence of specialized delivery techniques.

Despite the dramatic efficacy of the immune checkpoint inhibitors (CPIs) targeting CTLA-4, PD-1, and LAG-3 in cutaneous melanoma, similar efficacy has not been observed in UM. Several small retrospective studies have found evidence of limited activity of ipilimumab in UM, with response rates ranging between 0 and 5% and an OS of less than 10 months. Nivolumab, pembrolizumab, and atezolizumab are anti-PD-1 receptor or anti-PD-1 ligand (PD-L1) antibodies approved for the treatment of CM [10, 11]. Several retrospective studies have assessed the role of anti-PD-1 directed therapy in metastatic UM. In the largest of these studies, 2 out of 56 patients attained PR (3.6%) and 5 had stable disease (SD) (8.9%). Median PFS and OS were 2.8 and 7.6 months, respectively [12]. The reported results achieved with combined checkpoint blockade with ipilimumab and nivolumab are more promising, with synergistic response rates of 11.5–18% and a 1-year OS of greater than 50%. Given these low response rates with systemic CPI therapy, more active therapeutic approaches are needed. The proclivity of UM metastases for growth in the liver has stimulated interest in regional infusion approaches. While hepatic arterial infusion of therapeutics for UM liver metastasis has shown promise and is predicated on sound rationale, the choice of therapeutic agent is critical and should be tailored to the biologic drivers of disease progression within the liver, which include immunosuppressive cells such as myeloid-derived suppressor cells [4–6].

In this special issue of Cancer Gene Therapy, several groups present novel and innovative strategies for the treatment and diagnosis of metastatic UM. Quite a few promising translational and clinical strategies are presented, which focus on biologic features distinct to UM and the immunologic milieu in the liver. Wei and colleagues discuss targeted and epigenetic strategies which seek to leverage molecular targets associated with vulnerable disease-specific mechanisms. Orloff and colleagues review bi-specific therapies, including the recently approved agent, tebentafusp, which represents the first regulatory success in this disease and proof of concept that immunotherapeutics have the potential to drive better outcomes in metastatic UM. Aliahmad and colleagues discusses self-replicating RNA vectors for vaccines and immunotherapies, which offers the potential of a new therapeutic platform. Guha and colleagues examine the role of TLR9 inhibition in a murine model of liver metastases to eliminate myeloid-derived suppressor cells and promote a more immuno-responsive tumor microenvironment. A novel imaging based diagnostic strategy is presented by Yang and colleagues, who present an interesting study using chemokine receptor 4-based MRI imaging in a murine model of liver metastasis. Finally, Sheth and colleagues explore the role of regional therapeutic approaches in addressing the dominant site of failure in uveal melanoma patients—the liver.

While patients with metastatic UM continue to represent a population in need of better outcomes, meaningful progress has been made with respect to deepening our understanding of the disease biology and development of novel therapeutics, including the first regulatory approval of a systemic agent shown to improve survival. Innovative molecular and immunotherapy approaches that leverage biologic vulnerabilities in UM cells and specific immunosuppressive pathways driving disease progression in the liver may support further advances. We hope this special issue of Cancer Gene Therapy proves to be a valuable resource for those in the scientific and medical communities committed to improving UM patient outcomes.
